# Boas Práticas em Cardiologia – Uma Lição a Partir dos Indicadores de Desempenho

**DOI:** 10.36660/abc.20230033

**Published:** 2023-03-27

**Affiliations:** 1 Hospital de Clínicas de Porto Alegre Porto Alegre RS Brasil Hospital de Clínicas de Porto Alegre, Porto Alegre, RS – Brasil

**Keywords:** Doenças Cardiovasculares, Síndrome Coronariana Aguda, Insuficiência Cardíaca, Qualidade da Assistência em Saúde, Recursos em Saúde, Atenção à Saúde

A Organização Mundial da Saúde^1^ considera qualidade na assistência como “… o grau em que serviços de saúde para indivíduos e populações aumenta a probabilidade de desfechos de saúde desejados e que sejam consistentes com o conhecimento profissional baseado em evidências.” Além disso, considera serviços de saúde de qualidade aqueles que são efetivos, eficientes, seguros, equitativos e centrados nas pessoas.^1^

Visando o aumento da qualidade no cuidado cardiovascular em hospitais públicos brasileiros surge o Programa Boas Práticas em Cardiologia (BPC),^2^ iniciativa da Sociedade Brasileira de Cardiologia (SBC) em parceria com o Ministério da Saúde do Brasil e apoio da *American Heart Association* (AHA) e o Hospital HCor, através da adaptação do programa da AHA intitulado *Get With The Guidelines*^®^ (GWTG).^3,4^ O programa brasileiro é centrado nas doenças cardíacas de mais elevado custo, como a síndrome coronariana aguda (SCA), fibrilação atrial e insuficiência cardíaca (IC). Ele busca reduzir a mortalidade hospitalar, melhorar os processos hospitalares, – com foco na segurança e qualidade assistencial ao paciente cardiológico – e também reconhecer como centros de excelência em cardiologia os hospitais que atinjam as metas propostas. A métrica de avaliação deste programa baseia-se em identificar a taxa de adesão dos profissionais de saúde às recomendações das diretrizes da SBC e da AHA no tratamento destas três doenças cardíacas. Além disso, analisar o efeito sobre os desfechos tempo de internação, mortalidade por doença cardíaca, mortalidade por todas as causas, reinternação, qualidade de vida e percepção de saúde dos pacientes antes e após a implementação do programa.^2^

Contribuindo com esta iniciativa, o estudo de Passaglia et al.,^5^ se debruça sobre dados de 1036 pacientes adultos internados com diagnóstico primário de SCA e IC no período de 2016 a 2019 em um hospital público terciário de Minas Gerais, onde o Programa BPC foi aplicado.

Embora neste estudo as taxas de adesão global dos profissionais assistenciais aos indicadores de desempenho estabelecidos nas diretrizes da SBC e AHA terem sido altas e semelhantes, tanto no tratamento da SCA (92,9%), quanto no tratamento da IC (91,2%), se faz necessário escrutinar cada indicador. Na SCA, dos 8 indicadores de desempenho propostos, 7 foram avaliados (a terapia de reperfusão adequada não pode ser aferida). Destes, 6 (aspirina precoce, controle da pressão arterial e, na alta hospitalar, aspirina, inibidor da enzima conversora de angiotensina – IECA ou bloqueador do receptor de angiotensina – BRA, betabloqueador e estatina) apresentaram taxa de adesão acima de 85,0%, *benchmark* estabelecido pelo Programa BPC. O indicador de aconselhamento para parar de fumar ficou em 81,5%. Em relação ao tratamento da IC, dos 5 indicadores de desempenho previstos, somente 3 (avaliação de função ventricular esquerda por ecocardiograma, betabloqueador na alta hospitalar e consulta pós-alta hospitalar) tiveram taxa de adesão acima de 85,0%. Os outros 2 (IECA ou BRA e espironolactona na alta hospitalar) ficaram em 82,7% e 70,9%, respectivamente. Ou seja, abaixo do patamar preconizado. A taxa de óbito na internação ficou em 2,9% dos 763 pacientes com SCA e em 17,9% dos 273 pacientes com IC. Os dados evidenciados por este estudo demonstram nitidamente um espaço para aperfeiçoamento nos processos de cuidado e adesão às melhores práticas baseadas em evidências no tratamento da IC neste hospital terciário público.

Programas se propõem a melhorar a qualidade da assistência em hospitais públicos do SUS contribuem, de um lado, para a qualidade no atendimento e melhora dos desfechos e, de outro, não menos importante, para a redução da ineficiência e consequente mitigação de desperdícios financeiros de recursos escassos. A definição de indicadores, metas de desempenho e monitoramento fazem parte do arcabouço de conhecimento para a implantação de ações que visem à eficiência e qualidade da prestação de serviço em saúde.^6^ A relevância do estudo de Passaglia et al.^5^ vem corroborar a importância da utilização de métricas de avaliação e acompanhamento, como esta implementada pelo Programa BPC, que objetivamente explicitem deficiências e virtudes e contribuem com o aperfeiçoamento dos processos hospitalares e da qualidade na assistência em cardiologia.
