# Supplementary material for: Boas Práticas em Cardiologia – Uma Lição a Partir dos Indicadores de Desempenho
Source: Arq Bras Cardiol. 2023 Mar 27;120(2):e20230033. [Article in Portuguese] doi: 10.36660/abc.20230033 (PMC10473825; doi:10.36660/abc.20230033)
Supplement: Translation

# Translation: Good Practices In Cardiology – A Lesson From Performance Indicators

**Affiliations:** 1 Porto Alegre RS Brazil Hospital de Clínicas de Porto Alegre, Porto Alegre, RS – Brazil

**Keywords:** Cardiovascular Diseases, Acute Coronary Syndrome, Heart Failure, Quality of Health Care, Health Resources, Health Care (Public Health)

The World Health Organization^1^ considers the quality of care as “…the degree to which health services for individuals and populations increase the likelihood of desired health outcomes and are consistent with evidence-based professional knowledge.” In addition, it considers quality health services to be those that are effective, efficient, safe, equitable, and people-centered.^1^

Aiming to increase the quality of cardiovascular care in Brazilian public hospitals, the Good Practices in Cardiology Program (BPC)^2^ was created, an initiative of the *Sociedade Brasileira de Cardiologia* (SBC) in partnership with the Ministry of Health of Brazil and support from the American Heart Association (AHA) and the Hospital HCor, through the adaptation of the AHA program entitled Get With The Guidelines^®^ (GWTG).^3,4^ The Brazilian program focuses on the most expensive heart diseases, such as acute coronary syndrome (ACS), atrial fibrillation (AF), and heart failure (HF). It seeks to reduce hospital mortality, improve hospital processes – with a focus on safety and quality of care for cardiac patients – and also recognize hospitals that achieve the proposed goals as centers of excellence in cardiology. The evaluation metric of this program is based on identifying the adherence rate of health professionals to the recommendations of the SBC and AHA guidelines in treating these three heart diseases. In addition, to analyze the effect on the outcomes, length of stay, mortality from heart disease, all-cause mortality, rehospitalization, quality of life, and patients perception of health before and after the program implementation.^2^

Contributing to this initiative, the study by Passaglia et al.^5^ focuses on data from 1036 adult patients hospitalized with a primary diagnosis of ACS and HF from 2016 to 2019 in a tertiary public hospital in Minas Gerais, where the BPC Program implementation was applied.

Although in this study the overall adherence rates of healthcare professionals attending performance indicators established in the SBC and AHA guidelines have been high and similar, both in the treatment of ACS (92.9%), as in the treatment of HF (91.2%), it is necessary scrutinize each indicator. In ACS, of the eight proposed performance indicators, seven were evaluated (appropriate reperfusion therapy cannot be measured). Of these, six (early aspirin, blood pressure control and, at hospital discharge, aspirin, angiotensin-converting enzyme inhibitor - ACE inhibitor or angiotensin receptor blocker - ARB, beta-blocker, and statin) showed adherence rate above 85.0%, benchmark established by the BPC Program. The indicator counseling to stop smoking was 81.5%. Regarding the treatment of HF, of the five predicted performance indicators, only three (evaluation of left ventricular function by echocardiogram, beta-blocker at hospital discharge, and post-hospital discharge consultation) had an adherence rate above 85.0%. The other two (ACEI or ARB and spironolactone at hospital discharge) were 82.7% and 70.9%, respectively. Both are below the recommended level. The death rate during hospitalization was 2.9% of the 763 patients with ACS and 17.9% of the 273 patients with HF. The data evidenced by this study clearly demonstrate a space for improvement in care processes and adherence to evidence-based to the best evidence-based practices in the treatment of HF in this public tertiary hospital.

Programs that aim to improve the quality of care in hospitals in the Brazilian public health system (SUS) contribute, on the one hand, to the quality of care and improvement of outcomes and, on the other hand, not least, to the reduction of inefficiency and consequent mitigation of financial waste of scarce resources. The definition of indicators, performance targets, and monitoring are parts of the knowledge framework for implementing actions aimed at the efficiency and quality of health service delivery.^6^ The relevance of the study by Passaglia et al.^5^ corroborates the importance of using evaluation and follow-up metrics, such as the one implemented by the BPC Program, which objectively reveals deficiencies and virtues and contributes to the improvement of hospital processes and the quality of care in cardiology.
